# Multi-modal deep learning from imaging genomic data for schizophrenia classification

**DOI:** 10.3389/fpsyt.2024.1384842

**Published:** 2024-06-28

**Authors:** Ayush Kanyal, Badhan Mazumder, Vince D. Calhoun, Adrian Preda, Jessica Turner, Judith Ford, Dong Hye Ye

**Affiliations:** ^1^ Department of Computer Science, Georgia State University, Atlanta, GA, United States; ^2^ Tri-Institutional Center for Translational Research in Neuroimaging and Data Science (TReNDS), Atlanta, GA, United States; ^3^ Department of Psychiatry and Human Behavior, Univeristy of California Irvine, Irvine, CA, United States; ^4^ Department of Psychiatry and Behavioral Health, The Ohio State University, Columbus, OH, United States; ^5^ Department of Psychiatry, University of California, San Francisco, San Francisco, CA, United States

**Keywords:** schizophrenia, multi-modal, imaging genetics, deep learning, explainable artificial intelligence (XAI), single nucleotide polymorphism (SNP), functional network connectivity (FNC), structural magnetic resonance imaging (sMRI)

## Abstract

**Background:**

Schizophrenia (SZ) is a psychiatric condition that adversely affects an individual’s cognitive, emotional, and behavioral aspects. The etiology of SZ, although extensively studied, remains unclear, as multiple factors come together to contribute toward its development. There is a consistent body of evidence documenting the presence of structural and functional deviations in the brains of individuals with SZ. Moreover, the hereditary aspect of SZ is supported by the significant involvement of genomics markers. Therefore, the need to investigate SZ from a multi-modal perspective and develop approaches for improved detection arises.

**Methods:**

Our proposed method employed a deep learning framework combining features from structural magnetic resonance imaging (sMRI), functional magnetic resonance imaging (fMRI), and genetic markers such as single nucleotide polymorphism (SNP). For sMRI, we used a pre-trained DenseNet to extract the morphological features. To identify the most relevant functional connections in fMRI and SNPs linked to SZ, we applied a 1-dimensional convolutional neural network (CNN) followed by layerwise relevance propagation (LRP). Finally, we concatenated these obtained features across modalities and fed them to the extreme gradient boosting (XGBoost) tree-based classifier to classify SZ from healthy control (HC).

**Results:**

Experimental evaluation on clinical dataset demonstrated that, compared to the outcomes obtained from each modality individually, our proposed multi-modal approach performed classification of SZ individuals from HC with an improved accuracy of 79.01%.

**Conclusion:**

We proposed a deep learning based framework that selects multi-modal (sMRI, fMRI and genetic) features efficiently and fuse them to obtain improved classification scores. Additionally, by using Explainable AI (XAI), we were able to pinpoint and validate significant functional network connections and SNPs that contributed the most toward SZ classification, providing necessary interpretation behind our findings.

## 1 Introduction

Schizophrenia (SZ) affects approximately 1 in 300 individuals, with a global prevalence of around 24 million people, as reported by the World Health Organization (WHO) IHME (1). People with SZ could experience symptoms such as auditory and visual hallucinations, delusional thoughts, and disorganized thinking and behavior Frith et al. (2). The condition causes significant distress for the individual affected and also has an impact on their interpersonal connections, imposing a substantial burden on their personal and social life. In our society, SZ has a significant social and financial impact. People suffering from SZ often need medical attention, which further adds to these costs. SZ symptoms overlap with those of other mental disorders, making it difficult to precisely and promptly diagnose SZ Andreasen (3). The etiology of SZ, although extensively studied, remains unclear, as multiple factors come together to contribute toward its development. It is a complicated and heterogeneous neuropsychiatric disorder, that raises the necessity to study it from a multi-modal perspective. Our objective is to fulfill this goal by using a multi-modal approach where SZ is evaluated from morphological, connectivity, and gnomic standpoints. By effectively fusing features from these domains, an explainable deep learning-based framework was developed that is capable of representing this heterogeneous disorder better and can be used in further classification approaches.

Structural and functional deviations within the brain structure are significant factors in this contextHaukvik et al. (4). MRI scans of individuals with SZ exhibit subtle variations in brain structure and function, such as diminishing brain tissue and abnormal neural connections Lawrie et al. (5) Andreou and Borgwardt (6). There is a consistent body of evidence documenting the presence of structural deviations in the brains of individuals with SZ. However, these changes are non-specific and so subtle that they are easily overlooked by human experts. This raises the need for an automated diagnosis system that can analyze MRI data with meticulous precision and detect even the most subtle changes with high accuracy.

Several methods for the automatic classification of individuals with SZ Schnack et al. (7) SupriyaPatro et al. (8) using brain MRI had already been developed. One such method developed by Lung-Hao et al. Noble (9) used a support vector machine (SVM) based on brain-wise functional connectivity (FC) to classify SZ-affected individuals, and healthy controls based on the resting-state functional magnetic resonance imaging (rs-fMRI) Lee et al. (10). Wismuller et al. WismüCheck that all equations and special characters are displayed correctly.ller and Vosoughi (11) adopted an extended Granger causality technique to extract features from brain MRIs, which were then classified using SVM after identifying key attributes using Kendall’s tau rank correlation coefficient. However, these approaches frequently encounter difficulties in obtaining relevant features from brain MRIs. Deep learning has enabled the use of Convolutional Neural Network (CNN) architecture for automated feature learning from images Sadeghi et al. (12). Researchers used 3D sMRI data with deep learning models to collect significant spatial and morphological features Kanyal et al. (13)Mazumder et al. (14). In their study, Hu et al. used pre-trained 2D and 3D CNN models, as well as 3D sMRI technology Hu et al. (15), which aligned well with the “disconnection” hypothesis Frith et al. (16), which associated SZ symptoms with irregular neural connectivity networks and impaired cognitive process coordination. Data from functional brain connectivity had also been utilized with deep learning techniques to improve SZ classification outcomes. Phang et al. (17) Zheng et al. (18) Alves et al. (19) Oh et al. (20)

On the other hand, the hereditary aspect of SZ is supported by the significant involvement of genomics markers. Single-nucleotide polymorphisms (SNPs), which are variations in a single DNA nucleotide, have become important genomics markers that contribute toward the classification and explanation of SZ making genomics information useful in understanding and diagnosing SZ Corvin and Sullivan (21)Hannon et al. (22)Zamanpoor (23). SNPs reveal the unique variation in humans which can provide insights into the development of SZ Li et al. (24). However, given the large number of available SNPs, understanding all of the information they provide can be difficult. Moreover, employing all of these SNP variations as features can degrade the employed method’s performance Janecek et al. (25). Having too many features can be computationally expensive, and most SNP variations offer little to no insight into SZ. As a solution, deep-learning based methods can be employed to reduce data dimensionality while gaining a greater understanding of the genomic factors associated with SZ. To resolve this, we aimed to reduce the number of features employing explainable AI (XAI), which not only aided in the selection of the most relevant features but also ensured that the model’s output become easily interpretable. This is particularly important in high-stakes environments, such as medical imaging, where the accuracy and transparency of the model’s decision-making process are crucial and can help in the better overall diagnosis of disorder. Using XAI also helps to alleviate concerns regarding biased black-box deep learning methodologies and ensures that the model predictions are trustworthy, fair, and safe for use in critical applications Van der Velden et al. (26). Following that, as sMRIs, fMRIs, and SNPs all have distinct characteristics and provide complementary information, evaluating each of these elements separately generated three distinct latent spaces, which we fused through concatenation.

In summary, this work aims to identify biomarkers and improve prediction accuracies by fusing features from different modalities. Our approach models the SZ classification as a multi-modal problem which was analyzed with a multi-modal perspective that included three types of complementary data which provided a more extensive and nuanced understanding of the genomics and neural components that contributed to SZ while providing explainability behind our model’s predictions. Along with the explainability, the use of XAI helped significantly with feature selections, as using all available heterogeneous features from different modalities holds the potential to downgrade the model’s performance.

## 2 Materials and methods

**Figure 1:**
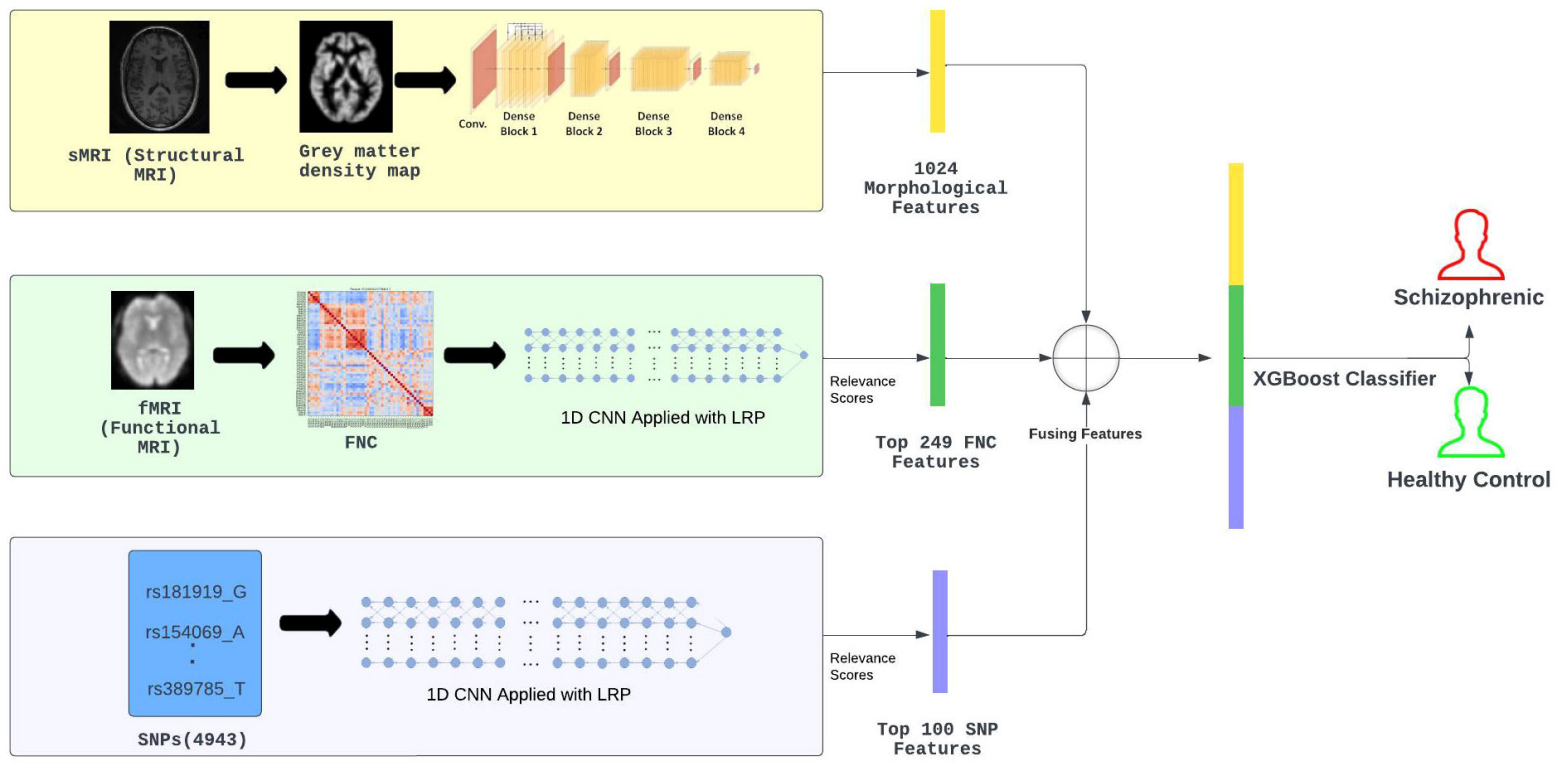
Method Overview: Initially, a memory-efficient, pre-trained DenseNet121 was used to extract morphological characteristics from 3D sMRI. Then, layer-wise relevance propagation following the 1D pre-trained CNN was utilized to select the top 249 FNC connections from all 1378 FNC connections and consider them as functional features. Similarly, the 100 most significant SNP features were selected using a pre-trained CNN with LRP. These features were then concatenated and fed into the XGBoost model for SZ classification.

In **Figure 1**, we present a visual overview and summary of our proposed multi-modal approach, leveraging various data modalities (sMRI, fMRI, and SNP), delineated into two prime stages.

In the first stage, feature extraction and feature selection were performed from different modalities. The morphological feature extraction was performed using a pre-trained denseNet121 model modified to work with 3D sMRIs. Next, the relevance scores of the 1378 functional connections were computed by considering the flattened lower triangular functional network connectivity (FNC) matrices. This was archived by utilizing layer-wise relevance (LRP) on a one-dimensional (1D) CNN that was specifically

trained on the lower-triangular matrix. Next, based on the relevance scores, the top 249 functional connections were selected to serve as functional features. Similarly, CNN was utilized to analyze genomic data which is represented as SNP, and using LRP the relevance score of each SNP was computed. Based on these scores, out of 4943 SNPs only a subset of 100 SNPs with the greatest relevance scores were selected to be used in our methodology. The selected SNPs served as the genetic features with the most genomic information for our classification task. Access to class labels (SZ diagnosis) for all the subjects in our experiments, meant all of our employed deep learning models were performing supervised learning tasks.

For the second stage, the selected features from sMRI, FNC, and SNP modalities were merged and following that the merged data was fed into an extreme gradient boost (XGBoost) Classifier. The motivation to use XGBoost for SZ classification came from past studies Gheshlaghi et al. (27) that successfully utilized transfer learning methodologies to categorize images of breast cancer histopathology. These investigations had shown that XGBoost is an effective classifier when dealing with multiple modalities of data sources. Moving forward, we provide a detailed description of the methods we used, diving deep into the intricate methods related to extracting features, determining relevance scores, and combining different types of data. The idea is to offer a clearer insight into how our proposed multi-modal deep learning approach works for SZ classification.

### 2.1 Dataset

For our experiments, a subset of the Functional Brain Imaging Research Network (FBIRN), The Bipolar and Schizophrenia Network for Intermediate Phenotypes (BSNIP), and the Center for Biomedical Research Excellence (COBRE) datasets were meticulously selected, encompassing participants diagnosed with schizophrenia (SZ) and healthy controls (HC). The dataset contains the sMRI, fMRI, SNP, and patient behavior of the subjects. For our investigation, the subjects were strictly limited within these cohorts having comprehensive data across sMRI, functional network connectivity(FNC), and genomics.

BSNIP Tamminga et al. (28) dataset is a collection of data from a network of laboratories striving to reveal phenotypes, genotypes, and biomarkers to elucidate psychosis. The consortium has made a dense phenotype process with the help of people who are grappling with psychosis all their lives, their family members, and healthy controls. This has created a database of biological information about people with SZ, schizoaffective disorder, and bipolar disorder with psychosis. The dataset encompasses 397 subjects diagnosed with SZ, possessing a mean age of approximately 35.7 years, among which there are 327 males. In comparison, the control group constituted of healthy individuals consists of 459 members exhibiting a mean age to around 36.5 years and includes 210 males. From BSNIP, a total of 96 healthy controls and 105 SZ-diagnosed subjects were included in our study with an average age of 36.37 years. Out of the selected subjects, there were 118 males and 83 females.

The FBIRN Keator et al. (29) dataset includes functional brain imaging data in addition to linked clinical and demographic information from participants. In the research cohort, 110 healthy controls and 76 patients with SZ were incorporated with an average age of 37.19 years. The age range of participants spanned from 18 to 62 years old. In the included subjects there were 146 males and 40 females. The repository contains structural and functional brain imaging data, clinical data, and cognitive data from people with SZ and HC. This dataset has been utilized for a broad variety of research purposes, such as examining the neuronal basis of various mental disorders, developing computational tools for evaluating brain data, and exploring biomarkers for detecting and comprehending brain-related conditions.

The COBRE provides raw anatomical and functional MR data from a total of 75 healthy controls and 72 patients diagnosed with SZ ranging in age from 18 to 65 years old in each group with an average age of 38.89 years. All of the individuals were screened, and those who had a history of certain conditions led to their exclusion from the study. These conditions included a history of neurological illness, a history of mental retardation, a history of serious head trauma with more than five minutes of loss of consciousness, and a history of drug addiction or dependency within the previous year. In the included participants there were 83 males and 22 females.

**Table 1** summarizes the dataset used in this study, including the source dataset from which the subset was obtained, along with the subjects in each class and their overall total. In total, our research dataset comprised of 492 participants, including 219 diagnosed with SZ and the residual group consisted of 273 healthy controls.

**Table 1:** Dataset description.

Datasets	Gender (Male/Female)	Age (Years)	Healthy Control	Schizophrenic	Total
FBIRN	146/40	37.19 ± 10.60	110	76	186
COBRE	83/22	38.89 ± 13.11	67	38	105
BSNIP	118/83	36.37 ± 12.27	96	105	201
Total	347/145	37.48 ± 11.99	273	219	492

### 2.2 Feature extraction

Prior to the utilization of compiled data in experimental trials, confirmation of some degree of standardization across datasets is imperative. The steps for pre-processing the raw data is replication from the work of Rehman et al. Rahaman et al. (30) where they used statistical parametric mapping and MATLAB-based tools.

sMRI is a three-dimensional (3D) map of brain morphology. The raw sMRI from all three datasets were processed using statistical parametric mapping (SPM12) in MATLAB using a reference brain image to spatially register our raw images. This was followed by joint bias correction, after which the brain tissues were categorically separated into white matter, grey matter, and cerebrospinal spinal fluid using which the

grey matter volume (GMV) was extracted. We selected the 3D grey matter density maps because it has strong spatial information that can be used to identify subtle alterations in the brains of SZ patients. Our processed output 3D grey matter density map had the dimensions of 121 × 145 × 121 voxels.

To extract the morphological features from the 3D sMRI, we used a pre-trained network called the denseNet121 Huang et al. (31) modified to work with 3D sMRI. Using denseNet, we were able to analyze the gray matter density maps that were obtained from sMRI. By using a pre-trained network, we adopted the transfer learning approach for extracting the morphological features.

The transfer learning approach uses the learning obtained from similar tasks to boost performance on a new task. This technique is valuable in analyzing medical images as it aids in overcoming the issue of medical data scarcity while also conserving time and computing resources Kim et al. (32). In relation to CNNs, transfer learning uses a pre-trained model and fine-tunes it for a specific task within medical imaging. The objective is to retain the fundamental characteristics comprehended by the model in its preliminary layers, and adjust subsequent layers congruent with our specific data and application scenario Kim et al. (32).

Transfer learning can be leveraged to extract features from the final layer of a pre-trained network and can also circumvent the issue of over-fitting. The application of transfer learning as a feature extraction strategy demonstrated enhanced performance on a restricted medical imaging dataset, specifically in the classification of breast histopathological images. Lang et al. (33)

In a standard CNN, the output from (*n* − 1)-th layer serves as the input to the (*n*)-th layer. This flow can be represented using $X^{\left(n\right)}=H_{n}(X^{\left(n-1\right)})$ , with $H_{n}$ denoting the non-linear transformation used at each step. This configuration of layers can cause an issue of vanishing gradients from the back layers to the layers at the front. Huang et al. (31) proposed DenseNet, which uses dense block structures, as a solution to this problem. These provide connections between every previous (*L*) layer and the current *n*-th layer in addition to its immediate predecessor (*n* − 1)-th layer, enabling efficient information transfer and preservation across all layers in our network as shown in Equation 1:


(1) $$X^{\left(n\right)}=H_{n}(\left[X^{\left(n-L\right)}+\cdots +X^{\left(n-2\right)}+X^{(n-10}\right])$$

The utilization of the configuration delineated in Equation 1 enhances inter-layer information transmission, consequently optimizing gradient flow. The 3D CNN utilizes a kernel of dimensions 7 × 7 × 7 and max-pooling to process the input data, which subsequently flows through the initial dense blocks. In these dense blocks, the number of layers is variable, and each of these layers comprises two 3D CNNs with distinct-sized kernels. The convolutional filters have dimensions of 1×1×1 and 3×3×3. The employment of the initial 3D-CNN results in a reduction in the dimensions of the feature map, thereby enhancing computational efficiency. The transition stage between the blocks consists of three distinct steps: first, the application of the ReLU activation function, then batch normalization, and last, reduction pooling between neighboring dense blocks. Subject-unique features are expressed using an output vector (*X_i_*) comprised of 1024 features, generated upon the application of a concluding global averaging layer Yu et al. (34).

Functional network connectivity (FNC) is a two-dimensional (2D) matrix representation of connectivity strengths between different regions of the brain. Functional magnetic resonance imaging (fMRI), a four-dimensional brain scan, measures changes in blood flow prompted by brain activity over time to produce the FNC. The fMRI was also pre-processed using the SPM12 Ashburner et al. (35) toolkit. To ensure that a steady state of magnetization was achieved, the initial five-time points from fMRI scans were disregarded. Thereafter, rigid body motion correction was performed to rectify any head movement by the subject. This was followed by slice-timing correction to address timing differences during slice acquisition. The brain can be divided into network components using Independent Component Analysis (ICA) Du et al. (36). Each component has its unique time course. FNC was calculated by measuring the changes among these component-time courses over time. This was done by calculating cross-correlations between the different brain networks (components). Calhoun et al. (37). As a result, we obtained a 53×53 diagonally symmetric 2D matrix for each subject depicting their functional network connectivity.

Due to the symmetrical characteristics of the FNC matrix, we extracted 1378 connections from its lower triangular matrix. The objective was to pinpoint the most crucial connectivity associations linked with SZ, employing these as connectivity features which significantly enhanced the model’s classification performence while minimizing time and computational resource usage. This was based on the idea that using a large number of features can adversely affect our model’s accuracy. A pre-trained one-dimensional CNNs was used specifically for carrying out layer-wise relevance propagation (LRP) on the FNC. In subsection 2.3, necessary details are provided regarding LRP. The application of LRP yielded relevance scores for each of the 1378 connections from the lower triangular matrix. On this, we performed feature selection by only keeping the top 249 connections that we utilized in our further analyses. The summation of all relevance scores yielded a total of 1. The selection process of the top 249 connections exclusively included features with relevance scores exceeding the threshold set at 0.002, identified through iterative experimentation and evaluation.

For the genetic data, the datasets were merged and preprocessed by the PLINK pipeline Purcell et al. (38). PLINK provides a robust and easy-to-use platform for executing numerous standard analyses using data from the entire genome. In our research, we shortlisted 4943 single nucleotide polymorphisms (SNPs) found within 108 risk Loci linked to SZ Chen et al. (39) Pantelis et al. (40). This was based on the findings from genome-wide association studies done by the Psychiatric Genomic Consortium for SZ. SNPs are a type of genomic variation that exists within the human genome. There are over 9 million SNPs documented in public SNP databases. These SNPs play an important role and act as key indicators in scientific research probing into the effects of variations in our genomes. Yang et al. (41) Kim and Misra (42) Aguiar-Pulido et al. (43) Zhao et al. (44) The genomic information is obtained through SNPs, which facilitates the differentiation between individuals with SZ and those who are healthy. The genomic data about SNPs is characterized by a higher dimensionality, with the majority of these SNPs being unrelated to SZ as noted in the study by Yu et al. Yu et al. (34). Therefore, it is essential to reduce the number of SNPs. A subset of 4943 SNPs associated with SZ risk was utilized, based on findings from the psychiatric genomic consortium. A one-dimensional 1D CNN was used in conjunction with layer-wise relevance propagation (LRP) to identify the leading 100 SZ-associated SNPs, which were subsequently utilized as the genomic features denoted as *W_i_*.

### 2.3 Explainable artificial intelligence (XAI) and feature selection

As we adopted a multi-modal approach in our work, the feature space can grow extremely large, and most of these features offer little to no insights into the pathways of SZ. The SNPs alone can provide tens of thousands of features that don’t contribute much to our model’s output. Along with that, there are functional connections too that have little influence over our model’s predictions. Thus, we needed a mechanism to perform feature selection, as processing a large feature set can be computationally expensive and could lead to the model making poor predictions rather than improving them. We overcame this challenge by employing XAI in our line of work. Employing XAI, we aimed to identify features from each modality that contributed the most to our model output. Following that we fused these features effectively to make the final predictions.

The purpose of explainable artificial intelligence (XAI) is to enable human experts to comprehend the underlying causes that led to the formation of an AI’s classification Pearl (45). XAI involves a set of procedures and methodologies that, when combined, allow users to understand and trust the results and outputs generated by machine learning algorithms Holzinger et al. (46). Most of the deep learning models are considered as black boxes that are impossible to comprehend Rocha et al. (47) Rudin (48). Deep learning utilizes neural networks, which are among the most complex and difficult for a human to understand. Chaddad et al. Chaddad et al. (49) have classified various AI techniques into ten categories: machine learning, neural networks and deep learning, data mining, knowledge discovery, and advanced analytics, rule-based modeling and decision-making, fuzzy logic-based approach, knowledge representation, uncertainty reasoning, and expert system modeling, case-based reasoning, text mining and natural language processing, visual analytics, computer vision and pattern recognition, hybridization, searching, and optimization. Several commonly used techniques in the field of explainable AI include feature importance analysis, rule extraction, surrogate models, visualizations, and counterfactual explanations. Feature importance analysis involves the identification of the most significant features within a model that contribute to its output. The process of rule extraction involves the extraction of rules from a model that is readily understandable to human beings. The concept of surrogate models involves the development of a simplified model that serves as an approximation for the behavior exhibited by a more complicated model. Visualizations involve generating visual depictions that portray data and models used within artificial intelligence systems. Counterfactual explanations generate hypothetical scenarios that could have resulted in different outcomes.

The core idea underlying the LRP algorithm for attributing relevance to individual input nodes is to trace back contributions to the final output node layer by layer Böhle et al. (50). The method of LRP has been established as a means of achieving explainable artificial intelligence it enables the decomposition of a deep neural network’s prediction over a sample, such as an image, into relevance scores for the single input dimensions of the sample, such as the pixels in an image Binder et al. (51). The proposed method uses a deep neural network’s back-propagation rule to calculate the relevance score for each feature for the classification outcome. The LRP is characterized by the preservation of information during the propagation process. Specifically, the neurons located in the lower layers of the neural network receive equal information from the upper neurons in a precise manner.

The characters *l* and *m* symbolize two neurons found in successive layers of the neural network. The function in Equation 2 shows the transmission of relevance scores $R^{m}$ from a given layer to neurons located in the previous layer:


(2) $$R^{l}=\sum_{m}\frac{z_{lm}}{\sum_{l}Z_{lm}}R^{m},$$

The variable $z_{lm}$ denotes the extent to which neuron *l* has contributed to the relevance of neuron *m*. The significance of the topmost layer can be found by computing the maximum value of the final activation. In order to effectively implement conservation, it is necessary for Equation 2 to maintain the constraint that $\sum_{l}z_{lm}$ remains constant, thereby ensuring that the overall level of relevance remains consistent as it transitions between layers. The propagation process is terminated when the next neuron is the input one. After applying the Equation 2 to individual neurons, it is crucial for the networks to maintain their layer-wise conservation property. The equation $\sum_{l}R^{l}=\sum_{m}Rm^{m}$ holds true. Upon the global application of the conservation property, it becomes possible to derive the rankings $R^{j},j=\;\{1,\cdots ,M\}$ for all *M*-dimensional input vectors from the model output.

The relevance score $R^{j}$ denotes the impact of each network connection in the SZ classification. The sorting of all 1378 functional network connections based on their relevance score lead to the selection of the top 249 FNCs associated with SZ as the connectivity feature $Z_{i}$ for a given subject *i*.

### 2.4 Data fusion and classification

At this stage we already obtained features from three different modalities ready to be fused and evaluated. To summarize, for each subject *i* we had 1024 morphological features, which can be denoted by *x_i_*, we used 249 functional network connections, denoted by *y_i_*, and 100 genomics features, denoted by *z_i_*. The fusion of these features is shown in the Equation 3:


(3) $$F_{i}=X_{i}⊕Z_{i}⊕W_{i},$$

Here, the ⊕ operator represents the vector concatenation.

We chose to use the XGBoost algorithm for our data classification due to its proven efficacy, as reported in previous studies Friedman (52) Livne et al. (53) Le et al. (54) Zhang et al. (55). XGBoost has been recognized for its exceptional capacity to tackle intricate issues in self-supervised learning involving large datasets. The architecture of the XGBoost combines both classification and regression trees (CART), resulting in a substantial acceleration of execution speed. By employing such a combination approach, it can address problems that would ordinarily be quite challenging with other classifiers. This model can predict the binary labels *y_i_* as shown below:


(4) $$y_{i}=\sum_{t}\;f_{t}\left(F_{i}\right),f_{t}\in \zeta$$

In Equation 4, *f* represents a functional domain function, *ζ* represents all possible CARTs and *t* denotes the number of trees. Given that individual CART parameters drive XGBoost parameters, identifying optimal parameter combinations during training sessions is crucial.

## 3 Results

This section goes over the experiments we conducted using our proposed deep learning based SZ prediction methodology. To assess the validity of our proposed method, we used 5-fold cross-validation with 80: 20 training testing split ratio. We also utilized this for tuning the parameters of the XGBoost classifier. Our experimental findings are further divided into quantitative (subsection 3.1) and qualitative evaluation (subsection 3.2) to explain our results in a broadly manner.

### 3.1 Qualitative evaluation

**Figure 2:**
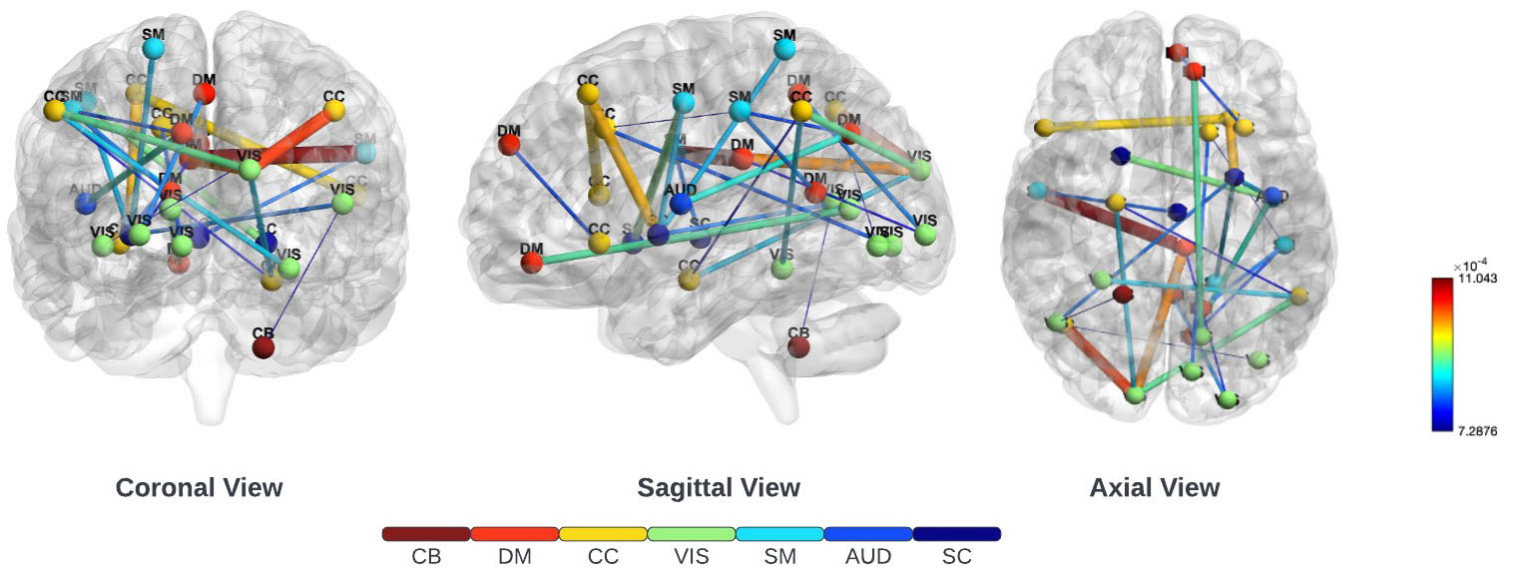
Three different views of the brain on the top 25 of 249 functional connections ranked by LRP. These included the subcortical (SC), auditory (AUD), sensorimotor (SM), visual (VIS), cognitive-control (CC), default-mode (DM), and cerebellar (CB) regions. LRP was employed on the lower triangle of the functional network connectivity matrix to find the most important 249 connections. Notably, there was a strong connection in the subcortical (SC) and sensorimotor (SM) areas, emphasizing their crucial significance.

To recognize and examine critical connections impacting the result within functional network connectivity, we used Layerwise Relevance Propagation (LRP). This method was applied specifically to the lower triangular matrix in identifying and extracting the top 249 crucial features from an initial pool of 1378 features. These were derived from all three employed different datasets: the Functional Biomedical Informatics Research Network (FBIRN), Consortium for Neuropsychiatric Phenomics (COBRE), along Bipolar-Schizophrenia Network on Intermediate Phenotypes (BSNIP) datasets. The top 25 of 249 functional network connections between various regions of the brain used in our study are illustrated in **Figure 2**. It is important to note the connections observed in both the subcortical (SC) and sensorimotor (SM) areas, underscoring their vital importance. Furthermore, the connections with the highest relevance scores were found in the sensorimotor (SM) and default-mode (DM) regions. This observation was consistent with clinical research that indicates the disruption of interactions between subcortical and cortical regions as the cause that underlies SZ Karbasforoushan and Woodward (56) Wheeler et al. (57) Hummer et al. (58).

In addition, our approach successfully identified SNPs linked to SZ, with particular emphasis on rs217291, rs217290, rs3798869, and rs217289 as the top four most significant genomic indicators. This discovery provides strong support for the findings of genome-wide association studies (GWAS) Dennison et al. (59), further emphasizing the significance of genomic makers in classifing individuals with SZ from HC.

### 3.2 Quantitative evaluation

To perform a thorough quantitative evaluation, we meticulously assessed the efficacy of our performed binary classification. **Table 2** summarizes our obtained results.

**Table 2:** Classification outcomes using 5 folds cross validation (mean ± standard deviation).

Modalities	Accuracy Scores	Precision	Recall	F1 Score
sMRI	0.6636 ± 0.06	0.6036 ± 0.14	0.6204 ± 0.13	0.6108 ± 0.13
SNP LRP	0.5706 ± 0.06	0.5929 ± 0.08	0.5319 ± 0.06	0.5581 ± 0.06
FNC LRP	0.7529 ± 0.02	0.7256 ± 0.04	0.6889 ± 0.04	0.7056 ± 0.02
sMRI + FNC (LRP) + SNP (LRP)	0.7901 ± 0.04	0.7883 ± 0.08	0.7943 ± 0.02	0.7893 ± 0.04

The XGBoost classifier attained a 66.35% accuracy rate solely by using sMRIs extracted morphological features, showcasing its efficacy in using sMRI data to differentiate between individuals with SZ and HC. Also, the XGBoost classifier displayed a notable 57.06% accuracy rate when using only SNP features, highlighting its competence in adeptly handling genomic data for classification tasks.

The XGBoost classifier demonstrated a noteworthy accuracy of 75.29% by using the 249 FNC connections that were chosen by the use of layer-wise relevance propagation (LRP). This points toward the significance of network connectivity in accurately distinguishing between SZ and healthy control subjects, thus highlighting it’s significance as a potential clinical biomarker.

The most significant performance improvement was observed after incorporating all three modalities. By effectively combining the 249 functional connections, 100 SNPs, and 1024 morphological features, we obtained improved performance of 79.01%, 78.83%, 79.43% and 78.93% in terms of accuracy, precision, recall and f1-score, respectively. The use of multi-modal data emphasizes the combined influence of morphology, genomics, and connectivity, providing strong evidence for the effectiveness of our proposed framework.

This study reveals the substantial benefits of a comprehensive approach that combines and integrates morphological, genomic, and connectivity data. By merging these three different domains, a significant performance gain had been obtained in terms of accuracy, precision, recall and f1-score when classifying SZ individuals, which may ultimately make a crucial contribution to the diagnosis and treatment of this neuropsychiatric disorder.

## 4 Discussion

Our obtained findings revealed the significance of our proposed novel multi-modal approach for SZ classification. The obtained performance gain in terms of all four evaluation indices provided persuasive evidence that functional network connections outperform both structural MRI and SNP as classification aspects. Significantly, the effectiveness of these functional network connections became particularly evident when compared to traditional sMRI and genomic data. Additionally, the classification performance tend to follow a steady growth pattern as we merged features from different modalities. There’s a significant boost in accuracy when two of three modalities fused features were employed compared to single modality features. Our proposed method, also effectively deals with limited training data and black-box issues of deep learning models. The employed explainability technique: LRP; performed feature selection and provided verifiable insights with clinical findings. Such as the connections between the SM and DM regions had the highest relevance score and the top four SNPs identified using LRP were also consistent with genome-wide association findings. These meticulously selected features provided significant performance gain during our downstream SZ classification task. The unique distinction of this approach lies in its revelation that the fusion of these modalities— FNC, sMRI, and SNP leads to an improved degree of classification performance. In this study, the integration of multiple data modalities provided a significant gain for SZ classification, highlighting the synergistic potential of our approach. Our proposed approach thus, allows for a comprehensive analysis of SZ across multiple domains, thereby increasing the likelihood of identifying any latent markers that the sole use of a single modality could overlook along with providing improved classification performance.

To ensure the robustness of our study, all experiments were performed with 5-fold cross-validation. The process of fine-tuning the hyper-parameters for the XGBoost classifier was achieved by iteratively experimenting with different values on the validation set. The objective of this last fine-tuning process was to enhance the performance and accuracy of the classifier in its final classification task. As we move ahead with research, one essential aspect becomes prominent: the need to standardize magnetic resonance imaging (MRI) images derived from a range of datasets. This step can protect against any biases that might inadvertently creep into our model due to different image contrasts stemming from various imaging equipment. Implementing this preventive measure has the potential to yield more balanced results.

## 5 Conclusions

In this work, we introduced a deep learning based multi-modal framework that incorporated three different modalities (sMRI, fMRI and SNPs) and leveraged explainable AI to attain better classification performance while limiting computational resource usage for identifying SZ individuals from HC. Particularly, 1-D CNN with LRP was applied on FNCs and SNPs to reduce features by selecting based on their corresponding relevance scores that resulted improving the model’s final classification and use of computational resources. Experimental outcomes obtained by performing evaluation on three clinical datasets proved the robustness and superiority of our introduced multi-modal approach.

## Data availability statement

Publicly available datasets were analyzed in this study. The FBIRN dataset can be found at https://www.ncbi.nlm.nih.gov/pmc/articles/PMC4651841/, with more information at https://www.na-mic.org/wiki/FBIRN:Main_Page; the BSNIP dataset can be found at https://www.ncbi.nlm.nih.gov/pmc/articles/PMC3934403/, with more information at https://nda.nih.gov/edit_collection.html?id=2274; and the COBRE dataset can be found at https://fcon_1000.projects.nitrc.org/indi/retro/cobre.html.

## Author contributions

AK: Writing – original draft, Visualization, Validation, Software, Methodology. BM: Writing – review & editing, Visualization, Software. VC: Writing – review & editing, Visualization, Investigation. AP: Writing – review & editing, Data curation. JT: Writing – review & editing, Data curation. JF: Writing – review & editing, Data curation. DY: Writing – review & editing, Validation, Supervision, Project administration, Methodology, Funding acquisition, Conceptualization.
